# Tuning Amorphous Selenium Composition with Tellurium to Improve Quantum Efficiency at Long Wavelengths and High Applied Fields

**DOI:** 10.1021/acsaelm.3c00150

**Published:** 2023-05-03

**Authors:** Kaitlin Hellier, Derek A. Stewart, John Read, Roy Sfadia, Shiva Abbaszadeh

**Affiliations:** † Department of Electrical and Computer Engineering, University of California, Santa Cruz, California 95064, United States; ‡ Western Digital Corporation San Jose Research Center, San Jose, California 95119, United States; ¶ Department of Physics, University of California, Santa Cruz, California 95064, United States

**Keywords:** amorphous chalcogenide, amorphous selenium, selenium telluride alloys, photoconductor, Onsager quantum efficiency

## Abstract

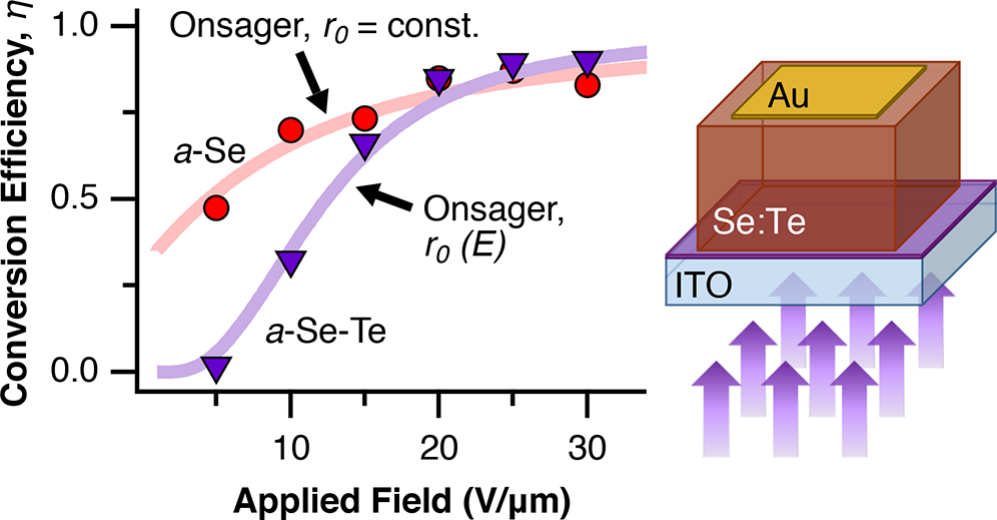

Amorphous selenium (a-Se) is a large-area compatible photoconductor that has received significant attention toward the development of UV and X-ray detectors for a wide range of applications in medical imaging, life science, high-energy physics, and nuclear radiation detection. A subset of applications require detection of photons with spectral coverage from UV to infrared wavelengths. In this work, we present a systematic study utilizing density functional theory simulations and experimental studies to investigate optical and electrical properties of a-Se alloyed with tellurium (Te). We report hole and electron mobilities and conversion efficiencies for a-Se_1–*x*_Te_*x*_ (*x* = 0, 0.03, 0.05, 0.08) devices as a function of applied field, along with band gaps and comparisons to previous studies. For the first time, these values are reported at high electric field (>10 V/μm), demonstrating recovery of quantum efficiency in Se–Te alloys. A comparison to the Onsager model for a-Se demonstrates the strong field dependence in the thermalization length and expands on the role of defect states in device performance.

## 1 Introduction

Amorphous selenium (a-Se) is a high-resistivity photoconductor that has many applications in X-ray detection, such as medical and industrial imaging, materials science, and threat detection. Amorphous Se is a leading direct-conversion photoconductive layer for thin-film-transistor (TFT) flat panel imagers and complementary metal-oxide semiconductor (CMOS) readouts used in detectors for mammography.^1−3^ Its high absorption and quantum efficiency (QE) in ultraviolet through blue wavelengths (∼80% at 400 nm and 30 V/μm) also make it well suited as a photodetector, with additional interest for the life sciences, high energy physics, and nuclear radiation detection.^4−7^ Recent work also suggests its alloys may have promise as memory and selector elements in nonvolatile memory systems.^8,9^ Its fabrication is a mature technology, and its photogeneration efficiency was extensively studied during the 1960s and 1970s.^10^ It is capable of avalanche multiplication at relatively low fields compared to other common avalanche materials, such as amorphous silicon.^11^ In most of its applications, a-Se is fabricated as a stabilized alloy (containing 0.2% to 0.5% As and 5 to 20 ppm of Cl). Adding As is beneficial for preventing crystallization; however it generates deep hole traps. Chlorine, in ppm amounts, is added to compensate for these As-induced deep traps, though it increases the density of shallow trap states and slightly reduces hole mobility.^12,13^

**Figure 1:**
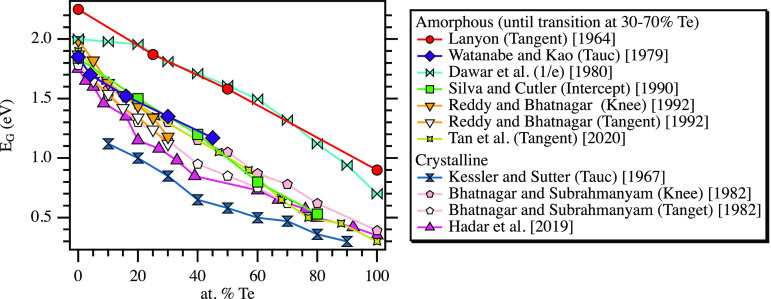
Reported Se–Te band gaps found in the literature for amorphous and crystalline films; the method used to calculate the gap is reported in parentheses in the legend.^14,15,19,20,22,24−27^

Indirect X-ray imaging (utilizing a scintillator coupled to the photodetector) and photodetection require a high QE in a greater range of wavelengths than a-Se can detect, extending into red and infrared wavelengths. Alloying a-Se with tellurium (Te) has been shown to decrease the band gap of the material, as demonstrated in Figure 1, and improves green to red absorbance.^14−16^ Glass transition and crystallization temperature, electrical transport, and optical and thermoelectric properties have also been shown to be tunable by alloying Se and Te at varying ratios.^14−24^ For example, an extremely low thermal conductivity of approximately 0.27 W/m·K was achieved for Se_0.15_Te_0.85_,^23^ and a responsivity of 1.5 A/W was achieved in Se_0.32_Te_0.68_ for short-wave infrared (1.55 μm).^24^

Despite the improvements seen in optical absorbance, studies have shown that the alloying of Te in a-Se results in a sharp reduction of carrier mobility due to the formation of defect states. Takahashi first found that the decrease in hole mobility saturates around ∼7%, transitioning from local to extended states and increasing electron trapping.^28^ He theorized that lone-pair electrons strongly interacted between Se and Te, with the fluctuation of these energy states giving rise to hole traps within the gap. Kasap and Juhasz later confirmed the reduction in mobility and provided additional evidence that Te induces both shallow and deep traps. The addition of Cl mitigates these deep traps much as it does in stabilized Se; however, Cl inclusion results in a further increase of shallow traps and thus a reduction in mobility.^17,18^ Polischuk et al. verified the formation of deep traps and the reduction in lifetime for a-Se_0.926_Te_0.074_.^29^ Reddy and Bhatnagar proposed that the formation of these defect states played a significant role in the reduction of the band gap with increasing Te content, offering several theories on the topic.^20^ Such large reductions in mobility and increases in defect states can be expected to have detrimental effects on the QE of Se–Te photoconductors.

Although a great deal of experimental data has been made available in alloying Se and Te for different applications, its charge transport has only been reported at low electric field (≤10 V/μm), possibly due to limits in instrumentation or dielectric breakdown, and little work has been done to understand how properties can be theoretically modeled and predicted. In this work, we present a systematic study utilizing density functional theory and hybrid functional (HSE) simulations and experimental studies to investigate the optical and electrical properties of alloyed amorphous Se–Te. We report carrier mobilities and quantum conversion efficiencies (QEs) for a-Se_1–*x*_Te_*x*_ (*x* = 0, 0.03, 0.05, 0.08) as a function of applied fields greater than 10 V/μm, along with band gaps and comparisons to previous studies. We find that, while the QE of Se–Te alloys is much lower than a-Se for fields below 10 V/μm, it can be recovered at fields above 15 V/μm. We explore the possible reasons for this, employing the Onsager theory for photogeneration, and find that a model utilizing a field-dependent thermalization length best fits experimental observations.

## 2 Results and Discussion

The role of Te in the reduction of the band gap of a-Se has been well studied experimentally; however, the modification of the density of states (DOS) has received less attention. Understanding how we can effectively simulate the DOS to predict behavior in new amorphous alloys is important for materials discovery; here we compare simulations of amorphous Se–Te with experimental observations.

**Figure 2:**
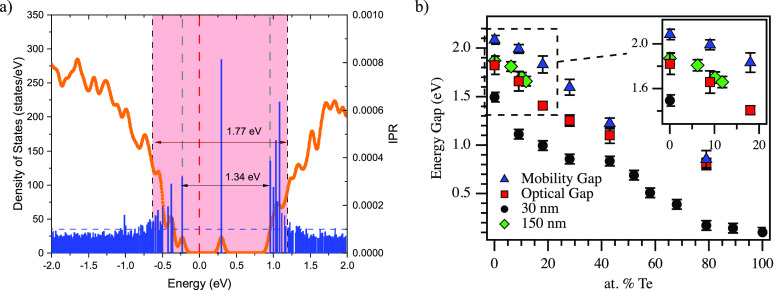
(a) The electronic density of states (orange trace) and the inverse participation ratio (IPR) (blue bar plot) are shown for a representative Se–Te alloy (Se_0.82_Te_0.18_). The Fermi energy for the system is denoted with a vertical red dashed line. The cutoff between localized and delocalized states (IPR = 1 × 10^–4^) is given by a horizontal blue dashed line and the two vertical black dashed lines, with the red-shaded region highlighting localized states. The predicted optical band gap (1.34 eV), the region between the highest occupied and lowest unoccupied states at the band edges and denoted with longer gray dashed lines, and mobility band gap (1.77 eV) are denoted by arrows on the figure. (b) Extracted values for the mobility and optical gaps from simulations (blue triangles and red squares, respectively) alongside the experimental results for optical band gaps calculated by the Tauc method for 30 nm sputtered and 150 nm evaporated amorphous Se–Te films (black circles and green diamonds, respectively).

The electronic DOS and inverse participation ratio (IPR) are shown in Figure 2a for a representative Se_1–*x*_Te_*x*_ (*x* = 0.18) alloy. Additional DOS and IPR plots for a-Se_1–*x*_Te_*x*_ (*x* = 0, 0.9, 0.28, 0.43, 0.79) can be found in the Supporting Information (SI). From the density of states, it is clear that the material possesses a band gap. The calculated DOS also shows the presence of defect states within the band gap. Using the inverse participation ratio, we can characterize the degree of localization for the energy states near the Fermi energy. The defect state roughly 0.3 eV above the Fermi energy, noted as a red dashed line in the figure, has the largest IPR, indicating a highly localized state. At the edge of the valence and conduction band, the states also exhibit significant IPR values and severe localization. The presence of localized states at the band edge is expected in the electronic structure of amorphous materials and is consistent with previous studies of Se–Te materials.^17,28,30^ For energies far from the band gap, the IPR is reduced and fairly uniform, indicating high-mobility carrier states. The optical band gap (1.34 eV) is measured as the energy difference between the highest occupied state and the lowest unoccupied state at the conduction band edge, highlighted by long gray dashed lines in the figure. Any defect states within the band gap are excluded from this analysis. The mobility band gap (1.77 eV) is determined by measuring the energy difference between the highest energy mobile occupied state and the lowest energy mobile unoccupied state. The transition from localized to mobile states is difficult to determine precisely. In this work, we are primarily concerned with material composition trends. We have assigned a state with an IPR value less than 1 × 10^–4^, indicated in the figure by dashed blue horizontal and black vertical lines, as being mobile and use this to determine the mobility band gap. Using the calculated DOS and IPR, we determined the optical and mobility band gaps for multiple configurations for several Se–Te alloy compositions, as can be seen in Figure 2b.

The predicted mobility and optical gaps decrease with increasing Te content, following trends observed in previous studies. With increasing Te content, the difference in mobility and optical gaps converges, indicating a reduction in the impact of optically active localized states near the conduction and valence band edges. Experimentally, it is known that Se–Te materials begin to exhibit some crystallization from 30% to 70%; this transition may contribute to the convergence in mobility and optical band gaps. For comparison of prediction to experiment, 30 nm films ranging from 0 to 100% Te and 150 nm films ranging from 0 to 12% were fabricated via sputtering and thermal evaporation, respectively, then characterized for optical transition energy. The experimental values extracted by the Tauc method are shown alongside the predicted energy gaps in Figure 2b. The observed optical band gap energy for all the 30 nm amorphous Se–Te alloys is lower than the simulated DOS values and those previously reported for bulk samples. The 150 nm samples, highlighted in the inset, fall in line with simulated gap values. The drop in observed band gap at the smaller thickness may indicate a thickness dependence potentially due to some quantum confinement effect or interfacial disorder. These issues are, however, outside the scope of this work. In both cases, the measured band gaps of the thin films show the trend of a reduced gap with increasing Te content, thus illustrating that the methods of calculation of DOS and IPR reported in this work provide a reliable format for the prediction of properties in future studies of alloyed-Se materials. The rate of decrease in the Se–Te alloys varies with increasing Te content, with a faster rate from 0 to 10% than 10–30% in sputtered films. This agrees with the trends reported by Reddy et al. for this range, with several possible explanations given, including a charged defect model, the role of localized states, and the effects of local short-range order.^20^

**Figure 3:**
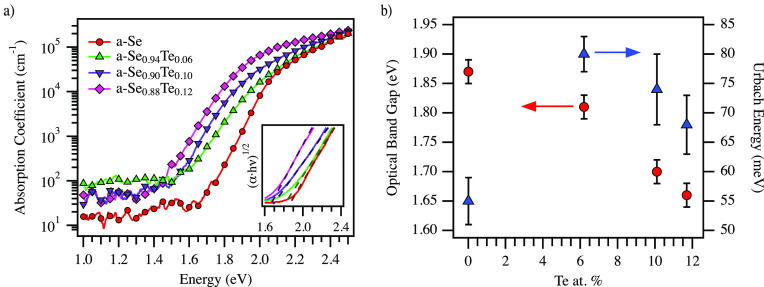
(a) Absorption coefficient of pure and a-Se_1–*x*_Te_*x*_ thin films (150 nm), as calculated from PDS measurements. Inset shows Tauc fits to the band edge region. (b) Optical band gaps, *E*_g_ (red circles, left axis), and Urbach energies, *E*_U_ (blue triangles, right axis), for 150 nm a-Se_1–*x*_Te_*x*_ films.

Our further studies focus on low Te content to increase absorption in a-Se while minimizing effects from increased conductivity and potential crystallization. The thicker of the thin films with ∼150 nm thickness were characterized using photothermal deflection spectroscopy to observe disorder in the alloys.^31,32^ The calculated absorption coefficient can be seen in Figure 3a, with Tauc fits shown in the inset. The shift of absorption to lower energies can readily be seen, along with an increase in tail states. Figure 3b reports the optical band gaps and the Urbach energy—a general measure of disorder—found for each sample. Urbach energies show an increase in disorder with the addition of Te, though we see an initial jump in *E*_U_ that reduces as the Te content increases. Reddy et al. proposed a band model in which the incorporation of Te leads to the rise of an additional optically active defect energy state just above the conduction edge.^20^ This would lead to a rapid increase in the tail absorption with Te inclusion and hence the increase in Urbach energy. As the gap decreases and shallow states begin to overcome the new defect state, the effect on the band edge disorder would be reduced, lowering *E*_U_, as we see from PDS fits.

**Figure 4:**
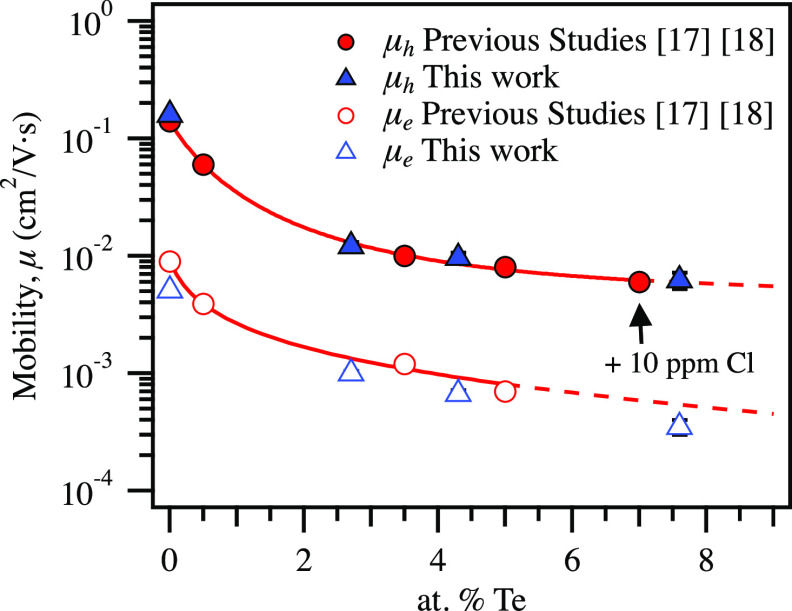
Comparison of hole (filled) and electron (unfilled) mobility at 5 V/μm for a-Se_1–*x*_Te_*x*_ devices fabricated in this work (blue triangles) to those of Kasap (1985) and Juhasz (1987) (red circles). The solid lines are inverse exponential fits to Kasap^17^ and Juhasz^18^ to provide a guide for the eye, with dashed lines the extension of the fit beyond available data.

To understand charge transport in these materials, 15 μm thick devices were fabricated and transient photocurrent time-of-flight was performed. The calculated hole and electron mobilities at 5 V/μm can be seen in Figure 4 and are compared with those found in previous studies.^17,18^ The hole mobilities found in this work are on par with those found in Kasap and Juhasz’s works, demonstrating that the alloys are behaving as expected. Alloying just a small amount (0.5 at. %) of Te results in a halving of the mobility, which can be detrimental to the performance of the device as a photodiode.

Electron mobility follows similar trends to hole mobility. It is important to note that, in contrast to previous studies and the standard for a-Se, the materials in this work were deposited at room temperature and not at 60–65 C, near the glass transition temperature. It is possible that this resulted in the slight reduction of the electron mobility of this work compared with Kasap and Juhasz, as seen in the plot.

**Figure 5:**
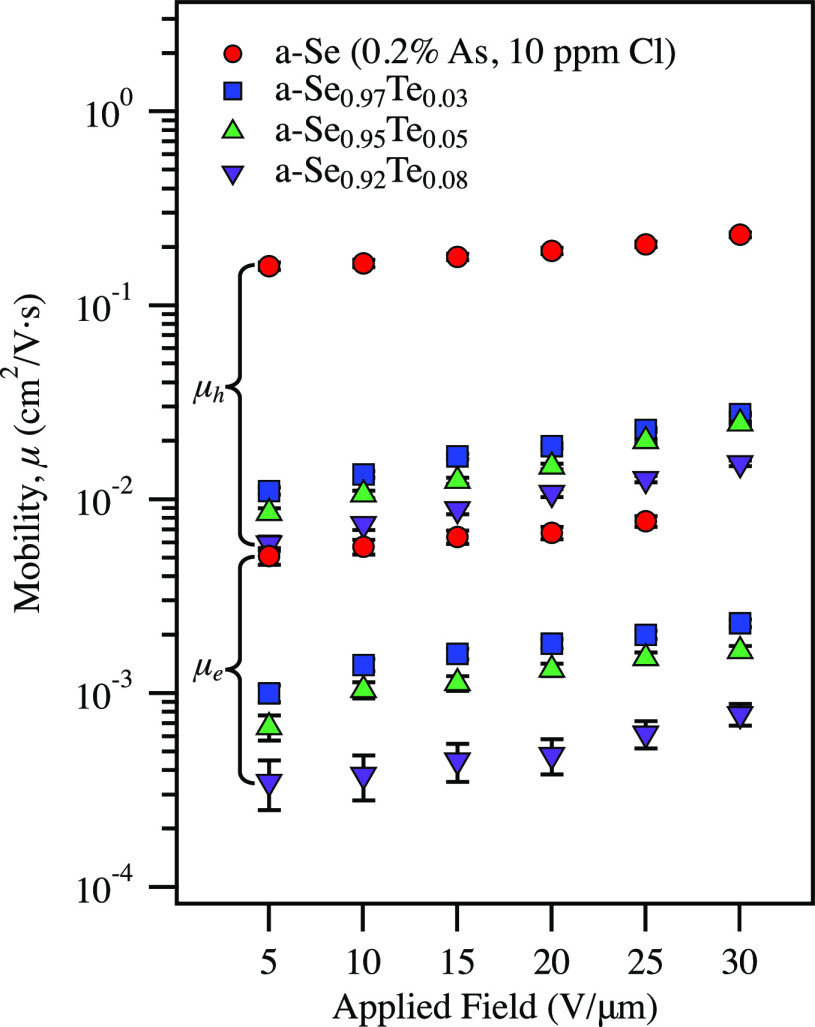
Hole and electron mobility as a function of electric field up to 30 V/μm for a-Se_1–*x*_Te_*x*_ devices fabricated in this work. Hole mobility is highlighted by the top bracket, and electron mobility by the bottom bracket.

The drop in both electron and hole mobilities has been investigated thoroughly in previous works; however, previous studies were typically performed on samples greater than 50 μm thick and were limited to fields of 10 V/μm or less. In this work, the use of thinner films allowed for probing up to 30 V/μm. Figure 5 shows hole and electron mobility for the a-Se_1–*x*_Te_*x*_ devices from 5 to 30 V/μm. Much like a-Se, the mobilities for the amorphous Se–Te devices increase at higher fields, though at a slightly higher rate.

**Figure 6:**
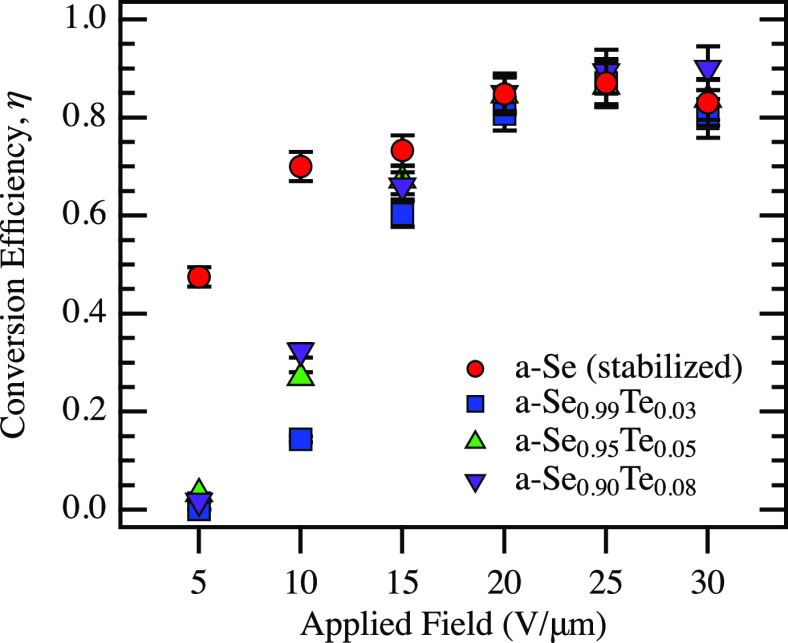
Conversion efficiency of a-Se_1–*x*_Te_*x*_ devices under 355 nm light as a function of electric field, up to 30 V/μm.

From TOF measurements, conversion efficiency at 355 nm was calculated for each device. Previous studies show that pure a-Se approaches efficiencies around 80% at 400 nm and 30 V/μm, agreeing within error with our results, reported in Figure 6.^5^ As may be anticipated from mobility measurements, the efficiency of Te-alloyed samples is much lower than a-Se at low fields; however, increasing the applied field has an increasingly positive effect, with Te-alloyed samples quickly approaching similar efficiencies to a-Se.

In amorphous selenium, it has been commonly accepted that the quantum conversion efficiency can be described using a model for electron–hole recombination originally proposed by Onsager.^33^ In this model, the incident photon leads to the creation of a bound electron–hole pair with some initial separation given as the thermalization length, *r*_0_. The electron–hole pair can then either recombine or else separate under the effect of the applied field and contribute to current. The charge motion in the Onsager model is treated as Brownian motion of the charge in the presence of the applied field and the Coulomb attraction due to the other photogenerated carrier. The quantum conversion efficiency then depends on both the efficiency of electron–hole creation under illumination and the probability that the generated electron–hole pair will dissociate. This approach was first used successfully by Pai and Enck to explain the photoconversion quantum efficiency in amorphous selenium as a function of applied fields for several different photon frequencies, each of which corresponds to a constant thermalization length.^5^

We apply this variant of the Onsager model in an attempt to explain the behavior observed in Se–Te devices. In their work, Pai and Enck developed a series expansion for the Onsager quantum efficiency that is slow to converge. Here, we have used the double integral expression developed by Yip et al., where the quantum efficiency, η, can be written as^34^
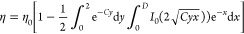
1where *C* = *eEr*_0_/*kT*, *D* = *r*_*c*_/*r*_0_, and *r*_c_ = *e*^2^/(4*πκε*_0_*kT*). In the equations above, η_0_ is the pair generation efficiency, taken to be 1, *I*_0_ is the modified Bessel function, *e* the fundamental charge, *E* is the electric field, *k* is the Boltzmann constant, *T* is the temperature, κ is the dielectric constant, ε_0_ is the permittivity of free space, and *r*_c_ is the critical separation distance. Results in this work were computed via Matlab. This model assumes that the thermalized electron–hole pairs have an initial separation length of *r*_0_. When fit with experiment, this separation length is found to correspond to a particular photon frequency, in which higher frequencies generate a greater separation and achieve higher conversion efficiency at lower fields.

**Figure 7:**
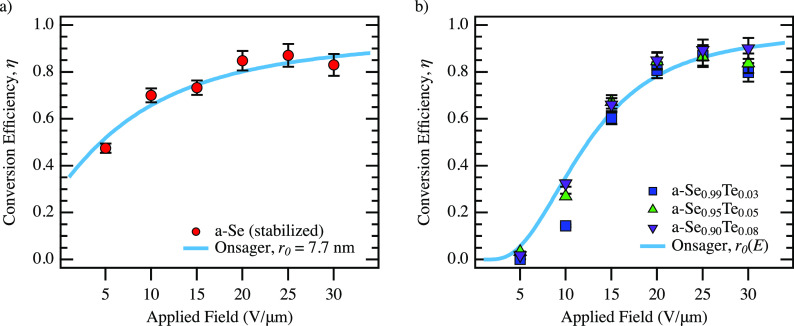
(a) Comparison between Onsager model and experimental results from the conversion efficiency of amorphous Se (355 nm incident light) as a function of electric field, up to 30 V/μm. (b) Comparison between Onsager model with field-dependent thermalization length and experimental results from the conversion efficiency of amorphous Se–Te samples (355 nm incident light) as a function of electric field, up to 30 V/μm. Fit used κ = 6.3, *r*_min_ = 0.1 nm, *r*_max_ = 12 nm, and γ = 4.5 × 10^–6^ cm/V.

We can use this equation to fit the conversion efficiency data for a-Se for λ = 355 nm incident light as a function of applied field. As shown in Figure 7, using η_0_ = 1, κ = 6.0, and *r*_0_ = 7.7 nm provides a good match to the experimental data. Pai and Enk determined the thermalization length for several different wavelengths of light in their study and also assumed that η_0_ = 1. If we extrapolate from their results to our smaller wavelength, their model would predict a larger thermalization length, 9.5–10 nm, greater than what we observe. This could be due to the fact that Pai and Enk used pure amorphous Se, whereas we have used stabilized amorphous Se in our study, or may be due to the reduced effect of photon energy on thermalization or relaxation at energies above a certain threshold.

Following a similar model, we attempted to describe the field dependence of the quantum conversion efficiency in the Te-doped devices. For these systems, we were unable to find a set of parameters that could explain the sharp increase in conversion efficiency with applied field. This likely indicates that the Onsager model is missing some key physics necessary to describe the efficiency of the Se–Te samples.

As discussed in several works, including Pai and Enck’s original paper, the thermalization distance is not well defined.^5,35−37^ Dependence of *r*_0_ on the material diffusion coefficient and mobility is known and is taken to be constant in Pai and Enck’s model. However, diffusion and mobility in a-Se and the Se–Te films vary with applied field, leading to the conclusion that *r*_0_ has field dependence.^12,38^ In addition, effects of large potentials from traps are not incorporated, and a simple form of binding energy is used in the original derivation.^5^

To address the issues of the fit, we initially assumed that thermalization length has an exponential dependence on field, *r* = *r*_0_ exp(*αE*). While using this mathematical form in the Onsager expression does lead to fits to the Se–Te sample results, the extracted thermalization lengths at higher fields are extremely large (practically infinite) and nonphysical.

To avoid this problem, we assumed that the thermalization length transitions smoothly from a short value at low fields to a larger value at high applied fields. This can be represented mathematically as
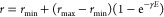
2

The comparison of the model with the field-dependent thermalization length and the Se–Te experimental results is shown in Figure 7. Overall, the Onsager model with the field-dependent thermalization length is able to describe the experimental trend observed in the Se–Te samples. It is important to note that for the doping range considered (3–8%), the field dependence of all samples is very similar, though we do see a (repeatable) increase in efficiency at lower fields with increasing Te content.

Amorphous selenium is known to operate by a multiple trapping transport mechanism, well described in ref (39). As previously discussed, Te dopants lead to the formation of additional trap states in the Se band gap. The addition of a new defect state, for which the energy above the valence edge shrinks with increasing Te, may be responsible for the strong field dependence of the charge dissociation and the slightly increased efficiency with higher Te concentrations for low fields. Carriers may initially have a low probability of escaping until the field strength has bent energy barriers enough to allow tunneling or hopping. This aligns with the observations made in Reddy and Bhatnagar and those made from the Urbach energies in this work.^20^ This indicates that the energy of the Te trap state dominates the effects associated with the concentration of the dopants, though higher concentrations may have benefits at low fields. Further study of concentrations, defect states, and temperature dependence may help to explore the role of Te in transport mechanisms for amorphous Se–Te alloys.

## 3 Conclusions

Alloys of amorphous Se–Te were fabricated and characterized for their potential as photodetector materials. Models evaluating the electronic density of states and inverse participation ratio demonstrated that the inclusion of Te reduces both the optical and mobility gaps, in agreement with observations in this and previous works. The incorporation of Te into a-Se results in an increase in Urbach energy, though increasing concentrations reduce this value; it is possible that this is the result of a defect state introduced by Te, in which the separation from the valence edge narrows with increasing Te concentration.

Hole and electron mobilities up to 8% Te show a drastic reduction compared with a-Se, in line with previous studies at low fields. We report these values up to 30 V/μm and see that mobility increases as a function of field, just as it does in a-Se. The conversion efficiencies at 355 nm are also reported as a function of electric field. At low fields, the efficiencies are significantly reduced with the inclusion of Te; however, the efficiencies increase with higher fields, eventually reaching values comparable to a-Se. Fits to the Onsager model suggest a highly field dependent thermalization length for Se–Te; the addition of a defect state above the valence edge may explain this and the resultant conversion efficiencies, providing further support for the model suggested by Reddy and Bhatnagar. However, further studies must be performed to obtain a full understanding of the role defect states and transport mechanisms play in these devices, especially at high fields. Future work will explore this while employing charge blocking layers, preventing dielectric breakdown at high fields and investigating the potential for avalanche multiplication. Regardless of the underlying physics, this work demonstrates the strong potential for amorphous Se–Te in extending the absorption range of a-Se photodetectors and expanding application in indirect X-ray imaging.

## 4 Experimental Section

### 4.1 Modeling/Simulation

First-principles density functional calculations were performed to understand how the electronic structures and band gap changes in Se–Te alloys as a function of composition. Simulations were done using the projector augmented wave approach included in the Vienna Ab initio Simulation Package (VASP).^40,41^ The Perdew–Burke–Ernzerhof (PBE) exchange–correlation functional based on the generalized gradient approximation was used to describe exchange and correlation energies in the system. In order to accurately capture the disorder inherent in an amorphous or glassy material, all simulations were done using supercells with 300 atoms. Comparison tests with supercells of smaller and larger sizes showed that this supercell size was sufficient to capture localization of electronic states in the band gap. For this study, 12 different alloy compositions were considered and the mass density for each composition was taken from the measured thin film values. Stochastic quenching was used to generate multiple initial configurations for each composition.^42−44^ For all configurations, structural relaxation was performed to ensure that atomic forces in the system were minimized. The total energies for the relaxed configurations at each composition show some distribution. However, this distribution is expected given that amorphous materials can have several metastable configurations.

Band gap predictions using density functional theory are well known to underpredict the experimental band gap and, in some cases, even predict that the system is metallic. Hybrid functional approaches like HSE06 that express the exchange–correlation energy in terms of contributions from density functional theory and Hartree–Fock exact exchange have been shown to provide reasonable estimates for the experimental band gap of many materials.^45^ For band gap predictions, we have taken the structures relaxed using density functional theory with the PBE functional and performed additional calculations using the HSE06 hybrid functional. The electronic structure of amorphous materials differs greatly from crystalline materials due to the loss of symmetry. In particular, the high degree of disorder leads to localized states both within the band gap and at the band gap edges. This disorder also pushes delocalized states capable of carrying current further away from the band gap center. In amorphous materials, this can lead to a significant difference between the optical band gap and the mobility band gap relevant for electronic transport.

For this work, we have calculated the electronic DOS to determine the position of states in terms of energy and combined this with a calculation of the IPR to distinguish between localized and delocalized energy states. The inverse participation ratio can be expressed in terms of the electron wave function, Ψ_α_ as
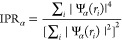
3

### 4.2 Fabrication

Amorphous films of selenium–tellurium alloys were fabricated by sputtering and thermal evaporation. Sputtered samples were deposited as reported in Read et al.^8^ Thermally evaporated samples were deposited as both thin (∼150 nm) and thick (∼15 μm) films for material and device characterization, respectively. In all cases, substrates were cleaned by ultrasonication in acetone and isopropanol for 10 min each, rinsed with DI water, and dried with nitrogen. Films were deposited in a dedicated selenium evaporator capable of uniform layers covering areas up to 4 in., with a deposition rate of ∼100 Å/s and with the substrate at room temperature and rotated at 40 rpm.^46^ Pure selenium (Sigma-Aldrich, 99.999%), stabilized selenium (0.2% As, 10 ppm of Cl, Amalgamet), and 10% and 20% tellurium-doped selenium (Amalgamet) were combined to achieve intended concentrations of 0–15% Te atomic weight. Thin film samples (100–200 nm) were deposited on 1 in. × 1 in. fused silica or indium tin oxide (ITO)-coated glass (Delta Technologies, Ltd.). Thick, or bulk, films were deposited on 1 in. × 3 in. ITO-coated glass and constructed into metal–semiconductor–metal (MSM) devices with the application of circular gold contacts, ranging from 3 to 5 mm in diameter, deposited by electron beam evaporation. Images of these films and devices can be found in Figure S1 of the SI.

### 4.3 Absorption Spectroscopy

The absorption coefficient of thin films was found from transverse photothermal deflection spectroscopy (PDS).^31,32^ Details regarding this technique may be found in previous works and in the SI.^47^ The band gap was calculated by the Tauc method, α ∝ (*h*ν·*E*_g_)^1/*n*^, where α is the absorption coefficient, *hν* is the optical energy, *E*_g_ is the energy gap of the semiconductor, and an indirect transition (*n* = 2) was assumed for all samples. The Urbach energy, *E*_U_, can be found by performing a linear fit in the Urbach region (just below the band edge) by *hν* ∝ *E*_U_ ln α.

### 4.4 Transport

Transient photocurrent time-of-flight (TOF) was carried out using a 355 nm, 25 ps pulsed laser (Ekspla) incident upon a device biased using an external high-voltage power supply (Weiner) and read out using a Keysight DSOS404A digital oscilloscope; carrier transit times, *t*_T_, were extracted from the resulting signal. Only a single pulse was used to generate the waveform, and laser intensities were kept as low as possible using UV neutral density filters in order to prevent distortion of the electric field from an abundance of carriers. Additional details can be found in the SI, along with a typical TOF waveform in Figure S6. Carrier mobilities were calculated using μ = *d*^2^/*V*_A_*t*_T_, where *d* is the material thickness and *V*_A_ is the voltage applied. Conversion efficiencies were calculated from TOF waveforms by integrating the signal and measuring the incident laser energy via a laser power meter and converting appropriately.

### 4.5 Amorphous Structure and Composition

X-ray diffraction was carried out on a Rigaku Miniflex II powder X-ray diffractometer at 3°/min with a step size of 0.02°, with a current and voltage of 15 mA and 30 kV, respectively. Cross-sectional scanning electron microscopy (SEM) of thin films on ITO was performed to determine the thicknesses of thin films, while stylus profilometry (Dektak-3) was used to determine bulk thicknesses. Energy dispersive spectroscopy (SEM-EDS) was conducted on all films on ITO by a Thermo Fisher Apreo SEM with an Oxford Ultim Max EDS operated at 15 kV, 1.6 nA to determine atomic ratios and confirm the homogeneity of Se and Te. Results of this work are included in the SI.

## Supporting Information Available

Images of fabricated films, a detailed schematic of the setup used for PDS, transient photocurrent TOF information, XRD data showing amorphous behavior, SEM-EDS data for composition and homogeneity, and additional DOS plots (PDF)
