# Supplementary material for: Tuning Amorphous Selenium Composition with Tellurium to Improve Quantum Efficiency at Long Wavelengths and High Applied Fields
Source: ACS Appl Electron Mater. 2023 May 3;5(5):2678–85. doi: 10.1021/acsaelm.3c00150 (PMC10210539; doi:10.1021/acsaelm.3c00150)
Supplement: Supplementary file 1 — el3c00150_si_001.pdf [file el3c00150_si_001.pdf]

Supporting Information
Tuning amorphous selenium composition with tellurium to improve quantum efficiency at long wavelengths and high applied fields

Kaitlin Hellier,[†] Derek A. Stewart,[‡] John Read,[‡] Roy Sfadia,[¶] and Shiva Abbaszadeh[*,†]

†*Department of Electrical and Computer Engineering, University of California, Santa Cruz*

‡*Western Digital Corporation San Jose Research Center,San Jose*

¶*Department of Physics,University of California, Santa Cruz*

E-mail: sabbasza@ucsc.edu

Film & Device Fabrication, Composition

Thin films of a-$Se_{1-x}Te_x$ (x = 0, 0.03, 0.05, 0.08, 0.11) and thick devices (x = 0, 0.03, 0.05, 0.08) were fabricated by thermal and e-beam evaporation. Figure S1 shows the four thin films studied, displaying a visible shift in color with increasing Te, and an a-$Se_{0.92}Te_{0.08}$ device, representative of all devices.

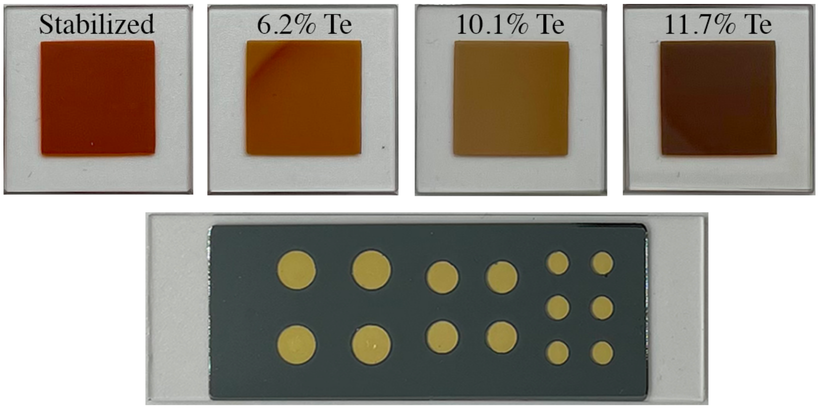

Figure S1: Images of thin films and an a-$Se_{0.92}Te_{0.08}$ device used in this study.

All films underwent SEM-EDS to determine the composition and ensure homogeneity of the Se-Te distribution. Compositional maps for the thin films can be found in Figure S2. The thicknesses of the thin films were found by cross-sectional SEM; thicknesses of the Se-Te layer of devices were found by profilometry. Values of Se and Te content and thickness can be found in Table S1.

Table S1: Actual thicknesses and compositions for thin film samples and devices. Calculated band gaps are given for the thin films

Sample	Thickness	Se	Te	E_g
a-Se	170 nm	100	-	1.88
a-$Se_{0.94}Te_{0.06}$	150 nm	93.8	6.2	1.72
a-$Se_{0.90}Te_{0.10}$	125 nm	89.9	10.1	1.64
a-$Se_{0.88}Te_{0.12}$	100 nm	88.3	11.7	1.62
a-Se (0.2% As, 10 ppm cl)	15.0 µm	100	-	
a-$Se_{0.97}Te_{0.03}$	14.3 µm	97.2	2.8	
a-$Se_{0.95}Te_{0.05}$	14.1 µm	95.3	4.7	
a-$Se_{0.92}Te_{0.08}$	14.7 µm	92.3	7.7	

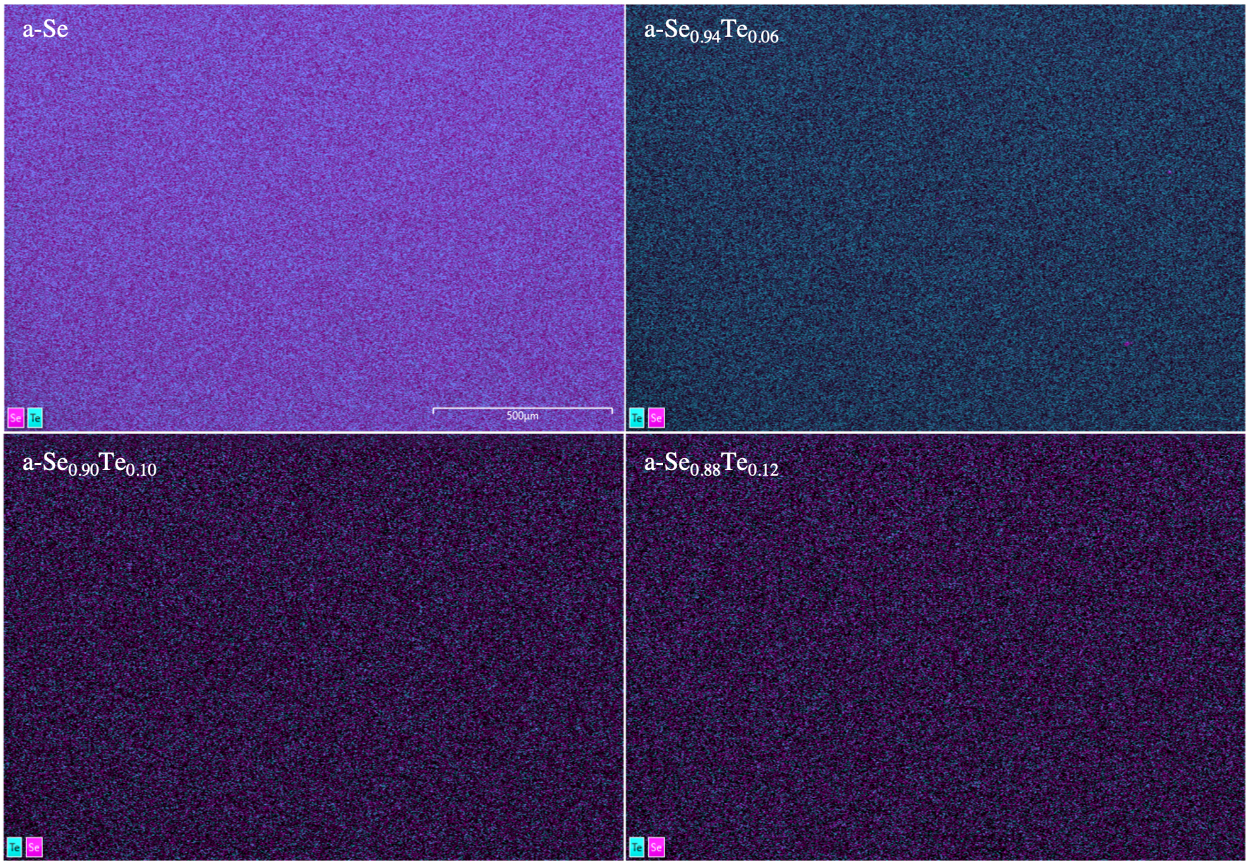

Figure S2: Compositional maps from SEM-EDS measurements showing a homogenous distribution of Se and Te in films.

An amorphous structure is important to the performance of the devices and ensures proper characterization of properties. X-ray diffraction was carried out on all films; Figure S3 shows scans of stabilized a-Se and a-$Se_{0.92}Te_{0.08}$, representative of all samples. The lack of sharp peaks and broad distribution around 25° is indicative of amorphous behavior.

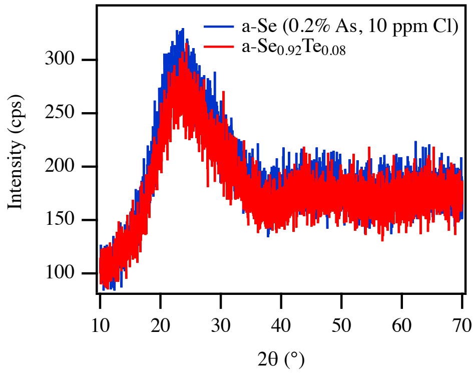

Figure S3: X-ray diffraction of stabilized a-Se and a-$Se_{0.92}Te_{0.08}$.

Photothermal deflection spectroscopy (PDS)

PDS is a high sensitivity technique for evaluating the non-radiative optical transitions below the band edge of a semiconducting material, down to 10 cm^{-1}. In the setup utilized in this work, a diagram of which can be found in Figure S4, the film is suspended in a liquid with a high gradient of index of refraction with respect to changes in temperature and pumped with a modulated (5 Hz) monochromatic beam (0.6-3 eV) normal to the sample surface. A HeNe probe laser is aligned adjacent and parallel to the sample surface; as the sample is excited and relaxes non-radiatively due to the modulated pump beam, the probe laser is deflected by the changing index of refraction of the suspension medium - in this case, Fluornert FC-72 (C_6F_{14}). This deflection is measured using a photodetector combined with a knife edge, measuring the change in the intensity of the laser incident on the detector. This signal, V_{sig}, combined with a reference of the pump intensity, V_{ref}, is used to calculate the absorption coefficient by

$$\alpha = \frac{-1}{d} \ln \left(1 - \frac{V_{sig}}{V_{ref}} C_{norm}\right) \tag{1}$$

with

$$C_{norm} = \frac{V_{ref}|_\lambda}{V_{sig}|_\lambda} (1 - T|_\lambda) \tag{2}$$

where d is the thickness of the film, and C_{norm} is a scaling constant determined by transmission, T, from UV-Vis spectroscopy at a wavelength just above the band edge. Final absorption coefficients were stitched together from UV-Vis measurements above the band edge and PDS measurements below the band edge. For additional information on the details of this system, please refer to.[1]

Transient photocurrent time of flight (TOF)

Transient photocurrent time of flight (TOF) is used to calculate the charge mobility in low-mobility materials, such as a-Se, by observing the transit time of carriers across the thickness of the device. In a vertical device, the absorption depth of the incident light is

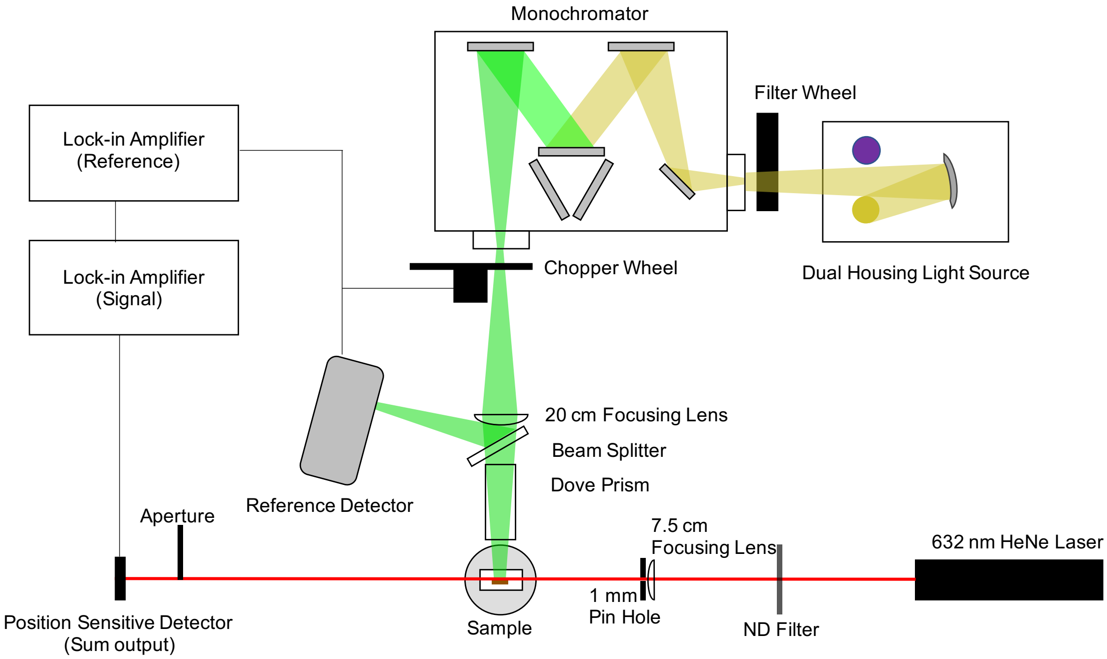

Figure S4: Birds-eye schematic of the PDS layout. Note that distances are not to scale or proportionally accurate.

much shorter than the thickness of the active layer; this generates charge carriers next to the transparent electrode. By biasing the device, one polarity of carriers is immediately collected at the transparent electrode, while the opposite carrier transits the length of the device and generates signal until collected at the opposite electrode. The time taken for charge carriers to transit the layer is used to determine the mobility of the carrier being measured.

A schematic of the TOF system used in this work is shown in Figure S5. The device is given a single pulse of 355 nm by a DPSS laser (EKSPLA), with a pulse width of 25 ps. A UV fused silica beam splitter (Thorlabs) splits the pulse between an energy meter and sample. Neutral density filters are used to ensure a small signal condition. Devices are biased using a high-voltage source. The signal from carrier transit is read out on a digital oscilloscope with 50 Ω impedance.

The mobility for each charge carrier is found by applying a positive (hole) or negative (electron) voltage to the ITO. Charge transit time, t_T, is found by taking the time between the onset of charge collection and the point at which lines fit to the plateau and tail intersect.

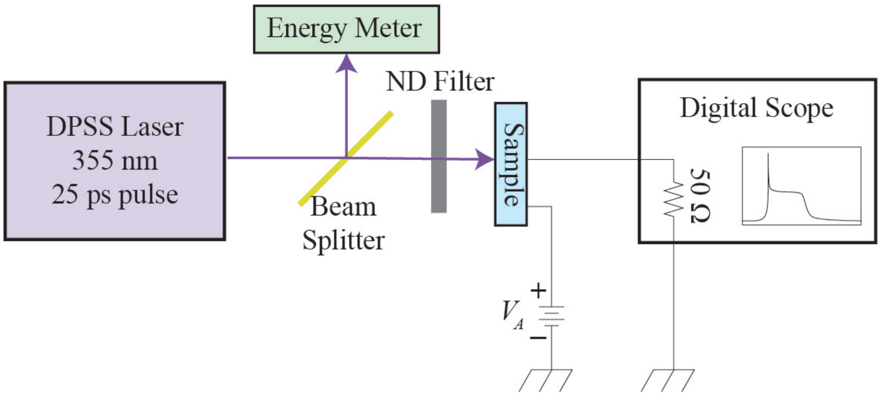

Figure S5: Schematic of the transient photocurrent TOF setup used in this work.

Mobility, μ, is then calculated as

$$\mu = \frac{L^2}{V_A t_T}; \tag{3}$$

where L is the thickness of the photoconductive layer and V_A is the applied voltage across that layer. Figure S6 gives an example of a typical TOF signal and the fits used to determine the transit time. A description of this system may also be found in.[2]

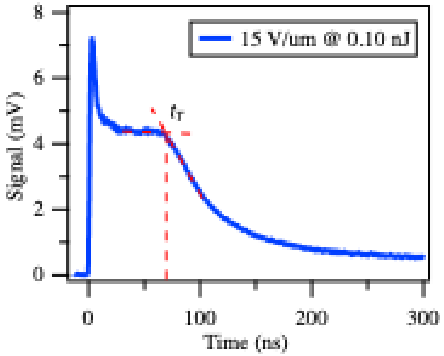

Figure S6: TOF signal of the 15 um vertical a-Se (0.2% As, 10 ppm Cl) device. Dashed red lines indicate how transit time was extracted.

DOS & IPR Plots

Density of states (DOS) and inverse participation ratio (IPR) simulations were used to predict the optical and mobility gaps of a-$Se_{1-x}Te_x$ alloys. Several iterations for each composition were performed to find an average value and error. One iteration from each composition can be seen in Figure S7; general trends in DOS and IPR values are consistent across itera-

tions, though locations of the defect states may vary.

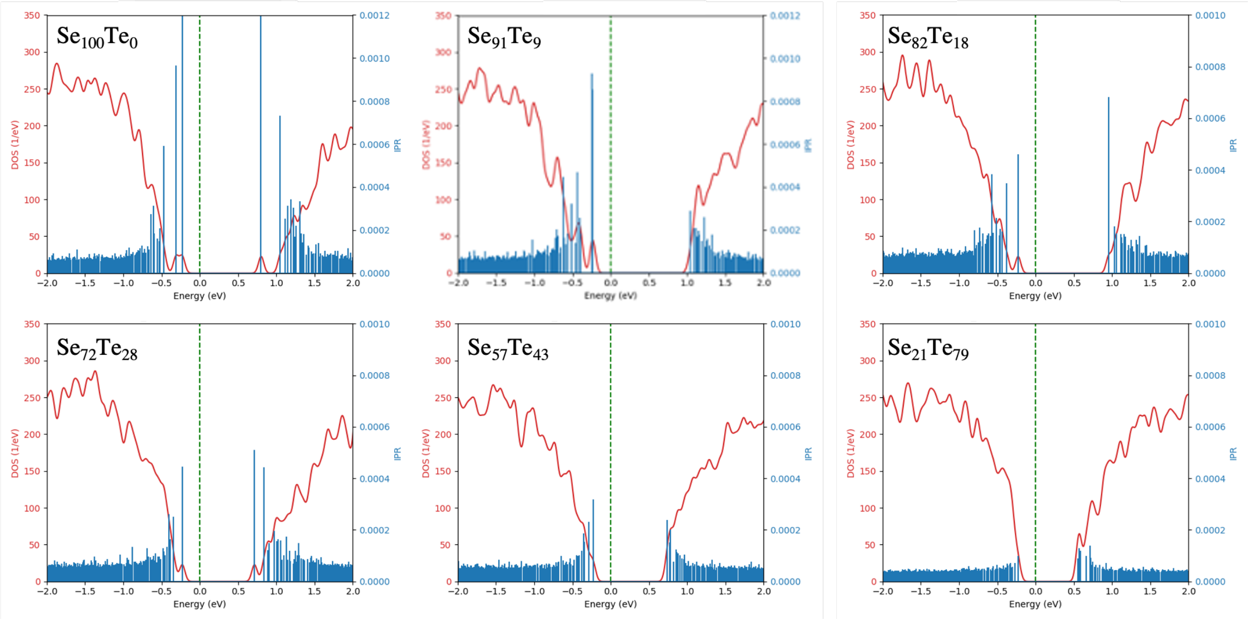

Figure S7: A DOS and IPR plot for each composition evaluated in simulations. Multiple iterations for each alloy were performed; plots are of one iteration, but show similar trends in DOS and IPR values across iterations.

References

(1) K. Hellier. Understanding the Impact of Local Structure on Materials for Optoelectronic Applications, University of California, Santa Cruz, 2020.

(2) K. Hellier, E. Benard, and S. Abbaszadeh. Tellurium Doped a-Se Devices for Improved Optical Absorption in Indirect X-Ray Photodetection, In Medical Imaging 2022: Physics of Medical Imaging, 12031:110–17. SPIE, 2022. https://doi.org/10.1117/12.2613150
